# Effects of a Rehabilitation Program Combined with Pain Management That Targets Pain Perception and Activity Avoidance in Older Patients with Acute Vertebral Compression Fracture: a Randomised Controlled Trial

**DOI:** 10.1155/2023/1383897

**Published:** 2023-02-13

**Authors:** Hideki Kataoka, Tatsuya Hirase, Kyo Goto, Yutaro Nomoto, Yutaro Kondo, Koichi Nakagawa, Junichiro Yamashita, Kaoru Morita, Yuichiro Honda, Junya Sakamoto, Minoru Okita

**Affiliations:** ^1^Department of Rehabilitation, Nagasaki Memorial Hospital, Nagasaki 851-0301, Japan; ^2^Department of Physical Therapy Sciences, Nagasaki University Graduate School of Biomedical Sciences, Nagasaki 852-8034, Japan; ^3^Department of Physical Therapy, Kanagawa University of Human Services, Yokosuka 238-0013, Japan; ^4^Department of Orthopedic Surgery, Nagasaki Memorial Hospital, Nagasaki 851-0301, Japan; ^5^Institute of Biomedical Sciences, Nagasaki University, Nagasaki 852-8034, Japan

## Abstract

This study aimed to investigate the effect of a rehabilitation program combined with pain management targeting pain perception and activity avoidance on multifaceted outcomes in older patients with acute vertebral compression fractures (VCFs). We randomised 65 older adults with acute VCFs to either an intervention group (*n* = 32), involving usual rehabilitation combined with pain management that targeted pain perception and activity avoidance, or a control group (*n* = 33), involving only usual rehabilitation. The usual rehabilitation was initiated immediately after admission. All patients were treated conservatively. Pain management aimed to improve the patients' daily behaviour by increasing their daily activities despite pain, rather than by focusing on eliminating the pain. Pain intensity and psychological statuses such as depression, pain catastrophising, and physical activity levels were assessed on admission. Two weeks postadmission and at discharge, physical performance measures were assessed along with the above-given measurements. A significant main effect of the group was observed for the intensity of lower back pain, favouring the intervention group (*F* = 5.135, *p* = 0.027). At discharge, it was significantly better in the intervention group than in the control group (*p* = 0.011). A time-by-group interaction emerged for magnification of the pain catastrophising scale (*p* = 0.012), physical activity levels (*p* < 0.001), and six-minute walking distance (*p* = 0.006), all favouring the intervention group. Rehabilitation programs combined with pain management targeting pain perception and activity avoidance could be an effective conservative treatment for older patients with acute VCFs.

## 1. Introduction

Fragility vertebral compression fractures (VCFs) due to osteoporosis have become an important health problem, owing to their increasing incidence in the aging population. A recent survey conducted in Japan reported an annual incidence of clinical VCFs diagnosed not only with imaging but also with clinical symptoms in people aged ≥65 years to be 15.58 per 1000 individuals [1].

Surgery is usually not required for all acute osteoporotic VCFs; hence, conservative treatment, comprising pain relief and rehabilitation is recommended [2]. Owing to a higher incidence of chronic pain due to VCFs and other health problems, effective pain management strategies for the acute phase are needed. Previous studies have indicated that 76% of patients with acute VCFs and treated conservatively experience severe pain even after one year, and 40% of patients continue to have disabling pain [3, 4]. Further studies have suggested that patients with VCFs are disabled and have lower activities of daily living (ADL) and quality of life (QOL) [3, 5]. Research findings from our previous study suggested that patients with persistent severe acute lower back pain, resulting from VCFs tend to be depressed and experience pain catastrophising, and persistent severe acute lower back pain, which negatively affects endurance and muscle strength but not ADL [6]. In addition, we previously indicated that lower physical activity during the acute phase leads to higher pain four weeks after treatment [7]. Therefore, pain management strategies for VCFs may require tailored approaches to address these factors.

Regarding pain management for VCFs during the acute phase, early mobilisation should be encouraged, as soon as it can be tolerated [8]. In addition, advice to remain active is the standard management for acute lower back pain [9]. However, owing to the low levels of physical activity observed in hospitalised older patients with VCFs [7], pain management strategies to increase physical activity are required. Furthermore, psychological techniques that alter dysfunctional ways of thinking, modify beliefs and attitudes and increase a person's control over pain and how they interpret pain are important for managing pain in older people [10]. Our previous randomised controlled trial demonstrated that exercise combined with pain management aimed at improving patients' daily behaviour by increasing their daily activities despite pain, rather than focusing on eliminating pain. This strategy effectively reduces pain intensity and improves psychological status and physical activity levels in older people with chronic musculoskeletal pain [11]. However, no study has demonstrated whether this pain management program, which targets pain perception and activity avoidance is effective in hospitalised older patients with acute VCFs.

This study aimed to investigate the effects of a rehabilitation program combined with pain management, targeting pain perception and activity avoidance on multifaceted outcomes in older patients with acute VCFs.

## 2. Materials and Methods

### 2.1. Participants

We enrolled patients with acute VCFs aged >65 years who were admitted to the emergency unit of Nagasaki Memorial Hospital, Nagasaki, Japan. Patients who were eligible for enrolment in the study were diagnosed with acute vertebral fractures based on acute pain in the back and lower back regions, a deformed vertebral body on radiography, and abnormal intensity within the vertebral bodies on magnetic resonance imaging. All fractures were confirmed to have originated from low-energy trauma. Patients were excluded if they were diagnosed with an unstable fracture, which affected all three columns (e.g., chance and burst fractures), were transferred from other hospitals, were unable to walk independently with or without a walking aid before hospital admission, unable to complete the questionnaire due to cognitive impairment, had a Mini-Mental State Examination (MMSE) score from 0 to 15, were defined as having moderately severe or severe cognitive impairment [12, 13], had complications that inhibited rehabilitation (for example, were haemodynamically unstable), had any other fracture type, were scheduled to be discharged within four weeks, or did not agree with the study protocol. The study protocol was approved by the Research Ethics Committee of the Graduate School of Biomedical Sciences of Nagasaki University (Approval no. 18110805). All the participants were informed about the study procedures and outcome measures and provided signed informed consent. This study was registered with the UMIN Clinical Trials Registry (UMIN000035426).

### 2.2. Design and Randomization

This randomised controlled trial was conducted between January 2019 and May 2021. The participants who met the inclusion criteria were randomised into two groups (1 : 1) using a computer-generated randomisation list. Block randomisation with a fixed block size of two was used to ensure similar sample sizes across the conditions. The groups included an intervention group, involving a usual rehabilitation program combined with pain management that targeted pain perception and activity avoidance, and a control group, involving only the usual rehabilitation program. An independent investigator (J. Y.) performed the randomisation after baseline assessment. A physical therapist in charge of each patient assessed the participants and implemented their usual rehabilitation and/or pain management programs.

### 2.3. Intervention

#### 2.3.1. Usual Rehabilitation Program

The usual rehabilitation program for patients in both groups was initiated immediately after admission (Table 1). All the patients were treated conservatively, as described in our previous study [6]. One week after admission, the patients were instructed to use a reclining bed and were permitted to move on the bed and walk around the room without a brace, but with the assistance of a healthcare worker to access the toilet. In the rehabilitation sessions, methods to reduce spinal pain during movement, such as turning over on the bed, were taught, and nonweight-bearing exercises of the upper and lower extremities were performed on the bed without bracing. After mild rehabilitation at the bedside, patients started mobilisation and exercises under bracing with a rigid or soft orthosis, which was adjusted according to the patient's injury level or with no orthosis. Rehabilitation after mobilisation included gait exercise, muscle strength training, balance exercise, and ADL exercises to prevent falls and allow a return home. The patients were instructed to wear the orthosis for 8–12 weeks. Furthermore, to manage acute low back pain, international guidelines recommend that general practitioners provide education and reassurance [14]. Thus, participants in both groups received education on the mechanism of VCFs and acute pain due to VCFs, conservative treatment and management of VCFs, and multifaceted pain.

#### 2.3.2. Pain Management Program

Patients in the intervention group also received a pain management program that targeted pain perception and activity avoidance one week after the start of rehabilitation (Table 1). The pain management program aimed to improve patients' daily behaviour by increasing their daily activities despite the pain, rather than focusing on eliminating pain. First, patients in the intervention group set goals for rehabilitation. Sharing decisions about rehabilitation goals with participants can have a positive impact on the patient's health and mental well-being [15, 16]; thus, rehabilitation goals were set based on shared decision-making, involving patients and physical therapists (H. K. and K. G.). Second, patients in the intervention group also underwent supervised physical activity using pedometers (Yamax Digiwalker SW-200; Yamasa Tokei Keiki Co., Ltd., Tokyo, Japan) and diaries. The patients were asked to wear the pedometer when awake and record their daily pain intensity, step counts, and behaviour in a diary [11]. Pain intensity was recorded using an 11-point numerical rating scale (NRS), ranging from 0 (“no pain”) to 10 (“worst imaginable pain”). Furthermore, our previous study revealed that the experience of successfully reaching step-count goals was useful in changing older participants' pain awareness [11]. Therefore, step targets were determined based on the first week and were calculated to increase the patients' average daily step counts by 5% in the second week, in accordance with previous studies [17]. Subsequently, step targets were calculated repeatedly and were increased by 5% every week until discharge if there was no worsening of pain or increase in fatigue. However, if the patients complained of worsening pain or increased fatigue, the step targets did not increase. Thus, the determination of step targets was based on shared decision-making, involving patients and physical therapists.

### 2.4. Assessments

#### 2.4.1. Baseline Characteristics

Data collected to characterise the patients at baseline included demographics, instrumental ADL before admission, and cognitive function. Patient demographics, including age, sex, height, body weight, body mass index (BMI), comorbidity, the locations and number of acute and previous fractures, and pain medication were obtained from the patients' medical records. Instrumental ADL before admission was assessed using the Tokyo Metropolitan Institute of Gerontology (TMIG) Index of Competence [18], and cognitive function was assessed using the Japanese version of MMSE [13]. Comorbidity was assessed using the Charlson Comorbidity Index (CCI), which includes 19 disease groups, where a higher score indicates a higher mortality risk [19]. The number of fractures was categorized into one, two, or more than two acute fractures, and zero, one, two, or more than two previous fractures, which is mild or more deformity according to the Genant classification [20].

### 2.5. Outcomes

Before beginning the study, physical therapists received training from one of the authors (H. K.), regarding assessment protocols. The primary outcome was lower back pain intensity during movements, such as rolling, standing up, and walking, which was assessed using an 11-point NRS [21]. Secondary outcomes were psychological status, physical activity levels, ADL, QOL, the ratio of the height of each border of the collapsed body to the posterior border of a normal upper vertebral body, and physical performance measures.

Psychological status, including depressive symptoms, pain catastrophising, and fear of movement or (re) injury was evaluated. Depressive symptoms were evaluated using the short Japanese version of the 15-item Geriatric Depression Scale (GDS-15) [22]. Pain catastrophising was evaluated using the Pain Catastrophising Scale (PCS) [23], which includes 13 items and three categories: rumination (five items), helplessness (five items), and magnification (three items). Fear of movement/(re) injury was assessed using the Tampa Scale for Kinesiophobia-11 (TSK-11) [24].

Physical activity levels were monitored using a uniaxial motion counter (Lifecorder GS®, Suzuken, Japan) placed at the right or left anterior superior iliac portion during admission (baseline). This motion counter can measure and store real-time estimates of the frequency, intensity, and duration of total physical activity [25]. The participants were asked to put on the motion counter whenever they were in the hospital, including during the therapy sessions, as well as in their bedroom. The motion counter was removed when the participant bathed or showered because it was not waterproof. Data from the motion counters were downloaded to the computer weekly to analyse the activity data (Y. K. and Y. N.). Using the motion counter, daily step counts and physical activity intensities were classified according to activity levels 1–9, and these activity levels were assigned to metabolic equivalents of 1.8–8.3. The average times of 1–9 (1.8–8.3) activity levels per week were recorded, according to our previous study [7].

We used the Functional Independence Measure (FIM) to determine the generic ability to perform ADL after admission. This performance-based disability tool assesses the disability level when performing basic ADL [26].

The EuroQOL 5-dimension3-level (EQ-5D 3L) was used to measure health-related QOL. The EQ-5D 3L descriptive system measures health-related QOL in five dimensions: mobility, self-care, usual activity, pain/discomfort, and anxiety/depression within three levels (i.e., no problems, moderate problems, and severe problems) and reports the result as a single five-digit number (from 11111, representing full health, to 33333) [27]. The single five-digit number can be converted into a single summary index value, which can range from −0.111 (for the Japanese population) to 1. Therefore, we adopted a single summary index value for statistical analysis in the present study, as in a previous study [28].

The collapsed vertebral body height was measured at the anterior (*A*), centre (*C*), and posterior (*P*) borders on lateral radiographs [29]. The posterior border height of the normal upper vertebral body (NP) was used as a reference. To evaluate fracture severity, the ratios of the *A*, *C*, and *P* border heights of the collapsed body to that of the NP were calculated as follows: (height of each border)/(height of NP) × 100%. Radiological measurements were performed by a physical therapist (H. K. or K. G.) who received specialised training from an orthopaedist (K. M.). Inter-rater (1, 1) and intrarater (2, 1) reliability values were high and acceptable, as described in our previous study [6].

Physical performance was evaluated using the chair stand test (CST), timed up-and-go test (TUGT), and 6-min walking test (6-MWT). During the CST, patients were asked to stand up and sit down, as quickly as possible five times, with their arms crossed in front of their chest. The floor-to-seat height is 45 cm [30]. The TUGT was performed by timing the ability of the patients to stand up from a chair, walk 3 m, turn around, walk back to the chair, and sit down [31]. The 6-MWT was used to assess whether functional exercise capacity was correlated with physical fitness. This test measures the distance (m) that a patient can walk on a flat and hard surface for 6 min [32].

Secondary outcomes, except for physical performance measures, were evaluated at admission, two weeks after admission, and at discharge. Physical performance was assessed two weeks after the admission and at discharge.

### 2.6. Required Sample Size

We used *G*^*∗*^ Power 333 to perform a preliminary test force analysis and estimate the required sample size[33]. The power was set at 0.8, and the significance level (a) was set at 0.05 [34]. The effect size for the two-way analysis of variance was set at 0.39, according to our previous study [11]. The power analysis indicated that 54 patients (27 per group) were required for pre- and postevaluations. We expected a 10% dropout rate [11], and required a minimum of 30 participants per group.

### 2.7. Statistical Analysis

We used the unpaired *t*-test or chi-square test to evaluate significant differences in patient characteristics before and after hospital admission between the intervention and control groups. The chi-square test was used for group comparisons of sex distribution and the proportion of dropouts. To analyse the effects of the rehabilitation program combined with pain management on outcome measures, analysis of variance (ANOVA) was used. A 3 × 2 [time (admission, two weeks postadmission, and at discharge) × group (intervention and control groups)] ANOVA for NRS, GDS, PCS, TSK-11, FIM, and physical activity, and loss of vertebral height was performed. A 2 × 2 [time (admission, two weeks postadmission, and at discharge) × group (intervention and control groups)] ANOVA for physical performance measures was also performed. Posthoc Bonferroni tests were used for specific comparisons, and significance was two-sided. All statistical analyses were performed using SPSS version 22 software (IBM Corp., Armonk, NY, USA).

## 3. Results

**Figure 1:**
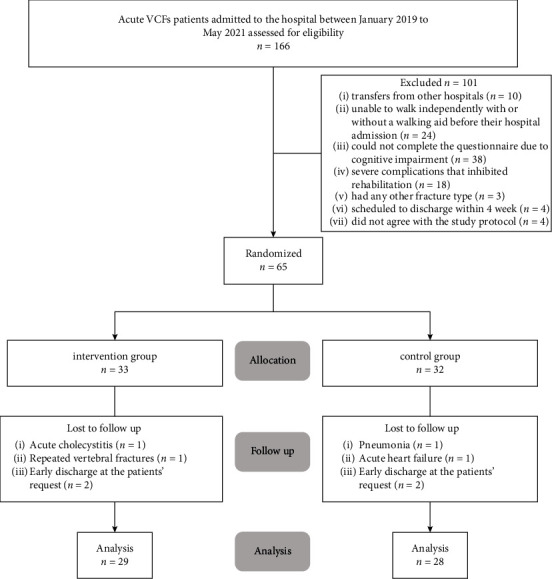
The flow of participants in the randomized trial.

Figure 1 shows a flowchart outlining the study participation. Between 24 January 2019 and 31 May 2021, we screened 166 potential participants aged ≥65 years who were admitted to the hospital with acute osteoporotic VCFs. Ten patients were transferred from another hospital to the Nagasaki Memorial Hospital after acute treatment. Twenty-four patients were unable to walk independently with or without a walking aid before hospital admission, and 38 could not complete the questionnaire because of cognitive impairment. Eighteen patients had severe complications that inhibited rehabilitation, and three had any other fracture type. Four patients were scheduled to be discharged within four weeks of admission, and four did not agree with the study protocol. Finally, we enrolled the remaining 65 individuals in the study and randomly allocated them to either the intervention group (*n* = 33) or control group (*n* = 32) (Figure 1). Eight (12.3%) patients withdrew from the trial; four dropped out of the intervention group, and four from the control group. There were no significant group differences in study withdrawal (*p* ≥ 0.99), and no patients dropped out because of the intervention program itself; 57/65 patients completed the intervention: 29 in the intervention group and 28 in the control group. For the intervention and control groups, the length of hospital stay was 51.1 ± 22.0 and 50.1 ± 20.5 days, respectively. There was no difference in the length of hospital stay between the two groups (*p*=0.863).

### 3.1. Baseline Characteristics

There were no significant differences in age, sex, height, body weight, BMI, CCI, TMIG Index of Competence before admission, location and number of acute VCFs, number of previous VCFs, pain medication, orthosis prescription, or MMSE scores between the intervention and control groups. No significant differences in pain intensity, psychological status, ADL, physical activity time, QOL, and the ratios of the *A*, *C*, and *P* border heights of the collapsed body to those of the NP were observed between the two groups (Table 2).

## 4. Effects of Rehabilitation Program Combined with Pain Management on Outcome Measures

In the intervention group, 26 patients (86.2%) achieved the target step count at discharge.  Table 3 shows the effects of the interventions on the outcome measures at all time points. In both groups, the mean NRS scores at two weeks and after admission and discharge improved significantly compared with baseline. A significant main effect of the group was observed (*F* = 5.135, *p*=0.027), and the mean NRS score at discharge was significantly higher in the intervention group than in the control group (*p*=0.011).

The mean PCS rumination, helplessness, and total scores at discharge in both groups improved significantly compared with the baseline, and there were no significant differences between the two groups at any time point. There were significant time-by-group interactions for PCS magnification (*p*=0.012), and the mean score at discharge in the intervention group improved significantly compared with the baseline. In both groups, the mean GDS-15 score at discharge improved significantly compared with the baseline, and there was no significant difference between the two groups at any time point. The mean TSK-11 score at two weeks and discharge in both groups improved significantly compared with baseline, and there was no significant difference between the two groups at any time point.

There were significant time-by-group interactions for daily step count (*p* < 0.001) and physical activity time (*p* < 0.001). In both groups, the mean step count and activity time at discharge improved significantly compared with those at two weeks after admission. Although we found no significant difference in the mean step counts and activity time two weeks after admission between the two groups, the mean values at discharge in the intervention group were significantly higher than those in the control group.

In both groups, the mean FIM score and EQ-5D 3L single summary index value at two weeks and discharge improved significantly compared with the baseline, and there were no significant differences between the two groups.

There were significant time-by-group interactions for ratios of the height of the C and P borders of the collapsed body to the posterior border of a normal upper vertebral body (*p*=0.006 and *p*=0.002, respectively), but not for the *A* border. In both groups, the ratios of the *A* and *C* border heights of the collapsed body to those of the NP at two weeks and discharge decreased significantly compared with the baseline. In the intervention group, the ratio of the *P*-border height of the collapsed body to that of the NP at two weeks and discharge decreased significantly compared with the baseline. We found no significant difference in the ratios of the *A* and *C* border heights of the collapsed body to those of the NP at two weeks and at discharge between the two groups.

The mean CST, TUGT, and 6-MWT values at discharge in both groups improved significantly compared with those at two weeks after admission. There were no significant differences in the mean CST and TUGT values at discharge between the two groups. There were significant time-by-group interactions for the 6-MWT (*p*=0.006), and the mean value at discharge in the intervention group was significantly longer than that in the control group.

## 5. Discussions

This study examined the effects of a rehabilitation program combined with pain management targeting pain perception and activity avoidance on multifaceted outcome measures in hospitalised older patients with acute VCFs. Currently, there is insufficient evidence regarding the use of conservative treatment for acute VCFs [35], and there are no universally accepted treatment methods for this condition [36]. Our rehabilitation program, combined with pain management, effectively reduced pain intensity, improved pain catastrophising, and increased exercise tolerance and physical activity levels. Thus, this intervention strategy could be an effective conservative treatment for older patients with acute VCFs.

In our pain management program, the patients' rehabilitation goals were first set based on shared decision-making. Systematic reviews investigating the effectiveness of goal setting in rehabilitation settings have shown that goal setting can improve patient adherence to treatment regimens; however, evidence for improved outcomes remains inconsistent [37]. In contrast, in patients undergoing a lower back pain rehabilitation program, goal setting has been found to have a positive effect on adherence to exercise and self-efficacy, but no effect on treatment outcomes in terms of physical function in rehabilitation settings [38]. Thus, goal setting may be effective in improving adherence to rehabilitation in patients in the intervention group.

Pain intensity, PCS magnification, and physical activity levels improved significantly in the intervention group compared to those in the control group. A systematic review revealed the effect of behavioural change interventions on physical activity levels in hospitalised patients, and the most common equipment used was accelerometers for feedback [39]. In addition, pedometers have been suggested as a successful motivational tool for increasing ambulatory activity in older adults [17]. Thus, our method would have been appropriate for older patients with VCFs to increase physical activity levels. Regarding acute pain and physical activity, it has been reported that patients who underwent lower limb arthroplasty with a substantial correlation between physical activity and postoperative day showed significant improvement in pain compared to patients with no correlation between them [40]. Furthermore, we previously reported that low physical activity during the acute phase is associated with higher pain at four weeks [7]. According to these previous studies, increasing physical activity levels is considered a key factor for improving pain in patients with VCF. In a review evaluating cognitive behavioural therapy, Hassett and Williams revealed that increasing physical activity levels is effective for managing chronic pain [41]. Furthermore, we previously investigated combined exercise and pain management that aimed to improve patients' daily behaviour by increasing their daily activities despite pain rather than focusing on eliminating pain, as advised by physical therapists for older people with chronic musculoskeletal pain [11]. Improvements in psychological status and physical activity levels have been reported [11]. It is indicated that professional feedback is important for increasing physical activity [42]; thus, our intervention using a pedometer and determination of step targets based on shared decision-making, involving participants and physical therapists, would be appropriate for patients with VCFs.

Although pain rumination and helplessness in both groups improved significantly compared with the baseline, pain magnification improvement was detected only in the intervention group. Pain magnification indicates a heightened perception of the threat represented by pain symptoms [43]. It has been suggested that the magnification component of catastrophising may be a risk factor for heightened pain in the acute phase [44]. In addition, pain magnification constitutes a psychological risk factor for chronic postsurgical pain in all surgical models [45]. However, a previous study revealed that an activity diary with goal setting during occupational therapy improved PCS magnification along with walking pain and physical activity levels in patients after total knee arthroplasty [46]. This result is consistent with our study; thus, improving the magnification of PCS would be one of the effects of pain management in this study, and it would be effective in preventing the transition from acute to chronic lower back pain.

The 6-MWT, identified as exercise tolerance, showed a better improvement in the intervention group than in the control group. Although no studies have investigated the effects of rehabilitation programs on exercise tolerance in older patients with acute VCFs, previous studies have indicated that promoting an increase in daily physical activity using a pedometer was effective in improving the 6-MWT in patients with the pulmonary disease [47, 48]. In these studies, not only the 6-MWT but also physical activity, such as step counts and improved significantly. Thus, increasing daily physical activity is important in improving exercise tolerance. In addition, we previously reported that persistent severe lower back pain during the acute phase of VCFs was associated with poorer 6-MWT results [6]; thus, improving exercise tolerance may be related to reduced pain, as noted in this study.

Considering the low withdrawal rates in the intervention group, it is suggested that the physical activity pacing approach using pedometers and a diary can be widely accepted by older patients with acute VCFs and is a safe and feasible pain management program. In addition, patients in the intervention and control groups had effectively improved pain, physical performance measures, ADL, QOL, and physical activity levels. These improvements are considered to result from the effects of the usual rehabilitation program, conservative treatment, and natural course of recovery from acute VCFs.

This study has several limitations. First, it lacks blinding. We could not choose patients blindly when deciding to assign them to either the intervention or control group, which was impossible because of the nature of the intervention. It was quite difficult to blind the physical therapists who intervened because the intervention was easily recognised. Furthermore, the same physical therapist assessed all patients who participated in the intervention program. Therefore, our results may have been influenced by physical therapists' expertise and/or reporting bias. However, all the physical therapists who participated in this study received a similar level of training before the study. Thus, we believe that the physical therapist's expertise had a minimal effect on this study. Second, in the intervention group, the ratio of the *P*-border height of the collapsed body to that of the NP at two weeks after admission and discharge decreased significantly compared with those at baseline. This indicates that the progression of the vertebral deformity was strong in the intervention group. However, we found no significant difference in the ratios of the *A*, *C*, and *P* border heights of the collapsed body to that of the NP between the two groups at any time point, and there were no adverse events, such as paralysis of the lower limbs. In addition, previous studies have revealed that the progression of vertebral body deformities is not associated with pain severity [6, 49]. Therefore, in this study, vertebral body deformities were considered to have no influence on pain. Third, because the study period was only during hospitalisation, the long-term effects of the intervention on pain, physical performance measures, and physical activity levels were not clear. Future studies with a longer followup period are required to investigate the effects of interventions on these outcome measures.

## 6. Conclusions

A rehabilitation program combined with pain management that targeted pain perception and activity avoidance significantly reduced pain intensity, improved pain catastrophising, and increased exercise tolerance and physical activity levels in hospitalised older patients with acute VCFs compared to the usual rehabilitation program. Our results indicated that this intervention program may be beneficial and could be an effective conservative treatment for patients with acute VCF. Furthermore, it may prevent the transition from acute to chronic pain.

## Figures and Tables

**Table 1:** Usual rehabilitation program and pain management program.

*Usual rehabilitation program (for the intervention and control groups)*	
Mild rehabilitation at the bedside (first week after admission)	(i) Bed mobility exercise and teaching the way to reduce spinal pain during movement
	(ii) Nonweight-bearing exercises of the upper and lower extremities were performed on the bed without bracing

Rehabilitation after mobilisation (second week after admission)	(i) After mild rehabilitation at the bedside, gait exercise, muscle strength training, balance exercise, and ADL exercise to prevent falls and allow a return home were performed

Education (from first to second week)	(i) About the mechanism of VCFs and acute pain due to VCFs, conservative treatment and management of VCFs, and multifaceted pain

*Pain management program (for the intervention group)*	

Aim	(i) To improve patients' daily behavior by increasing their daily activities despite pain, rather than focusing on eliminating pain

Goal setting	(i) Rehabilitation goals were set based on shared decision-making, involving patients and physical therapists

Physical activity approaches	(i) Pedometers (Yamax digiwalker SW-200; Yamasa Tokei Keiki Co., Ltd., Tokyo, Japan) and diaries were used
	(ii) Patients were asked to wear the pedometer when awake and record their daily pain intensity, step counts, and behaviour in a diary
	(iii) Step targets were increased by 5% every week if there was no worsening pain or no increase in fatigue
	(iv) Step targets were not increased if patients complained of worsening pain or increased fatigue

**Table 2:** Participants' characteristics and outcome measures at baseline.

Characteristics	Intervention group (*n* = 33)	Control group (*n* = 32)	*p* value
Age	81.1 ± 8.8	81.7 ± 8.4	0.7813
Female, *n* (%)	25 (75.8)	23 (71.9)	0.7218
Height (cm)	154.1 ± 8.0	153.9 ± 8.3	0.9344
Body weight (kg)	53.7 ± 12.4	53.2 ± 11.8	0.8868
BMI (kg/m^2^)	22.5 ± 4.5	22.4 ± 4.3	0.9018
Charlson comorbidity index	1.3 ± 1.3	1.3 ± 1.5	0.9805
TMIG Index of competence before admission	9.0 ± 0.6	8.0 ± 0.6	0.2269
Fresh VCFs location			
Th6-Th10/Th11-L2/L3-L5	3/29/6	5/26/7	0.6905
Number of fresh VCFs, *n* (%)			
One/Two/More than two	28 (84.8)/5 (15.2)/0 (0.0)	27 (84.4)/4 (12.5)/1 (3.1)	0.5729
Number of previous VCFs (%)			
Zero/One/Two or more	8 (24.2)/14 (42.4)/11 (33.3)	9 (28.1)/11 (34.4)/12 (37.5)	0.7997
Pain medications			
Loxoprofen-Na	8	9	0.7725
Celecoxib	9	12	
Acetaminophen	17	13	
Tramadol	5	4	
Orthosis prescription			
Soft/rigid/no	19/8/6	17/10/5	0.8151
MMSE	24.9 ± 2.3	24.3 ± 3.8	0.4107
NRS	7.8 ± 1.9	8.1 ± 2.0	0.6089
PCS			
Rumination	14.6 ± 4.0	14.2 ± 4.0	0.7179
Helplessness	8.5 ± 4.6	8.3 ± 5.5	0.8309
Magnification	5.2 ± 3.2	3.8 ± 3.1	0.0793
Total	28.3 ± 9.9	26.3 ± 10.8	0.4343
TSK-11	28.5 ± 5.4	29.0 ± 5.6	0.7175
GDS-15	6.0 ± 3.5	6.9 ± 3.1	0.2672
Daily step counts	401.7 ± 486.8	427.9 ± 393.9	0.8240
Physical activity time (s)	235.0 ± 333.5	269.2 ± 236.8	0.6598
FIM	67.8 ± 15.9	64.4 ± 19.7	0.4445
EQ-5D 3L	0.258 ± 0.253	0.297 ± 0.263	0.555

Ratios of the height of each border of the collapsed body to the posterior border of a normal upper vertebral body			

Anterior (%)	72.3 ± 16.2	72.0 ± 16.6	0.9507
Center (%)	60.4 ± 14.2	58.9 ± 13.6	0.6596
Posterior (%)	98.4 ± 9.0	95.3 ± 10.5	0.1849

Data are presented as median and mean ± standard deviation or number of patients (%). BMI, body mass index; VCFs, vertebral compression fracture; MNA-SF, Mini nutritional assessment short form; TMIG Index of competence, Tokyo metropolitan institute of gerontology index of competence; MMSE, mini-mental state examination; NRS, numerical rating scale; PCS, pain catastrophising scale; TSK-11, Tampa scale for Kineshiophbia; GDS-15, 15-item version of the geriatric depression scale; FIM, functional independence measure; EQ-5D 3L, EuroQOL 5-dimension3-level.

**Table 3:** Group comparison of outcome measures during the intervention period.

	Intervention group			Control group			Main effect				Time-by-group interaction	
							Group		Times			
Items	At admission (baseline)	Two weeks after admission	Discharge	At admission (baseline)	Two weeks after admission	Discharge	*F*-value	*p*value	*F*-value	*p*value	*F*-value	*p*value
NRS	7.9 ± 1.9	4.7 ± 2.0^‡^	1.7 ± 1.2^†,‡,§^	7.9 ± 2.0	5.2 ± 2.0^‡^	2.9 ± 2.1^‡,§^	5.135	0.027	131.371	≤0.001	1.388	0.254
PCS												
Rumination	14.4 ± 4.2	12.4 ± 5.2	8.3 ± 5.6^‡,§^	14.2 ± 3.8	12.7 ± 5.4	9.3 ± 6.0^‡,§^	0.113	0.738	32.224	≤0.001	0.343	0.710
Helplessness	8.4 ± 4.7	6.0 ± 3.6^‡^	4.3 ± 3.8^‡^	8.2 ± 5.3	6.0 ± 4.6^‡^	4.8 ± 4.5^‡^	0.012	0.913	14.681	≤0.001	0.162	0.851
Magnification	5.3 ± 3.3	3.8 ± 3.5^‡^	2.6 ± 2.8^‡^	3.5 ± 2.6	3.5 ± 2.9	3.3 ± 2.6	0.509	0.479	5.924	0.004	4.637	0.012
Total	28.1 ± 10.5	22.1 ± 10.7^‡^	15.1 ± 11.1^‡,§^	25.9 ± 9.7	22.1 ± 10.7	17.4 ± 11.6^‡^	0.000	0.996	25.684	≤0.001	1.107	0.334
TSK-11	28.4 ± 5.6	25.4 ± 6.4^‡^	22.8 ± 6.8^‡,§^	29.4 ± 5.0	25.5 ± 4.8^‡^	24.3 ± 5.9^‡^	0.440	0.510	25.032	≤0.001	0.404	0.669
GDS-15	6.2 ± 3.7	6.1 ± 4.3	4.1 ± 3.5^‡,§^	7.0 ± 3.1	5.9 ± 3.1	4.9 ± 3.6^‡^	0.263	0.610	12.700	≤0.001	0.890	0.414
Daily step counts	411.4 ± 493.3	820.7 ± 852.2	2866.4 ± 1666.7^†,‡,§^	427.1 ± 401.1	849.6 ± 1327.1^‡^	1570.2 ± 977.7^‡,§^	4.055	0.049	63.579	≤0.001	10.384	≤0.001
Physical activity time (s)	245.4 ± 344.3	506.3 ± 558.3	1831.1 ± 972.1^†,‡,§^	267.7 ± 241.2	572.7 ± 899.7^‡^	1095.5 ± 642.1^‡,§^	2.624	0.111	67.939	≤0.001	8.663	≤0.001
FIM	68.216.5	91.1 ± 18.0^‡^	116.1 ± 8.4^‡,§^	67.0 ± 16.8	90.3 ± 16.9^‡^	112.8 ± 8.0^‡,§^	0.297	0.588	314.331	≤0.001	0.263	0.769
EQ-5D 3L	0.265 ± 0.252	0.571 ± 0.205^‡^	0.741 ± 0.162^‡,§^	0.283 ± 0.272	0.637 ± 0.152^‡^	0.720 ± 0.192^‡,§^	0.257	0.614	106.288	≤0.001	0.917	0.403

Ratios of the height of each border of the collapsed body to the posterior border of a normal upper vertebral body												
Anterior (%)	73.0 ± 16.9	62.3 ± 16.6^‡^	53.6 ± 16.7^‡,§^	71.4 ± 16.8	65.4 ± 18.4^‡^	58.1 ± 19.2^‡,§^	0.245	0.622	60.917	≤0.001	2.269	0.108
Center (%)	60.9 ± 14.7	51.1 ± 15.0^‡^	42.4 ± 15.8^‡,§^	58.5 ± 13.9	54.0 ± 15.7^‡^	47.9 ± 16.9^‡,§^	0.316	0.576	68.519	≤0.001	5.253	0.006
Posterior (%)	98.4 ± 9.7	94.7 ± 10.0^‡^	89.7 ± 10.4^‡,§^	95.3 ± 10.6	93.7 ± 9.3	92.7 ± 9.8	0.018	0.894	20.396	≤0.001	6.503	0.002
CST (s)	—	19.8 ± 10.0^†^	12.8 ± 4.2^§^	—	17.4 ± 5.6	12.3 ± 3.0^§^	1.086	0.302	51.639	≤0.001	1.281	0.263
TUGT (s)	—	20.9 ± 12.4	11.8 ± 5.6^§^	—	19.5 ± 8.5	12.8 ± 4.7^§^	0.008	0.930	42.839	≤0.001	1.035	0.314
6-MWT (m)	—	209.7 ± 121.3	344.6 ± 113.4^†,§^	—	196.8 ± 105.9	261.3 ± 107.5^§^	3.149	0.082	66.262	≤0.001	8.259	0.006

Values are expressed as mean ± standard deviation. NRS, numerical rating scale; PCS, pain catastrophising scale; TSK-11, tampa scale for kineshiophbia; GDS-15, 15-item version of the geriatric depression scale; FIM, Functional independence measure; EQ-5D 3L, EuroQoL 5-dimension3-level; CST, chair stand test; TUGT, timed up-and-go test; 6-MWT, six-minute walk test. ^†^Significant between-group differences (*p* < 0.0167 for comparison of NRS, PCS, TSK-11, GDS-15, daily step counts, physical activity time, FIM, EQ-5D 3L, and loss of vertebral height, and *p* < 0.025 for comparison of physical performance). ^‡^Significant intragroup difference from baseline (*p* < 0.0167 for comparison of NRS, PCS, TSK-11, GDS-15, daily step counts, physical activity time, FIM, EQ-5D 3L, and loss of vertebral height; *p* < 0.025 for comparison of physical performance). ^§^Significant intragroup differences at two weeks (*p* < 0.0167 for comparison of NRS, PCS, TSK-11, GDS-15, daily step counts, physical activity time, FIM, EQ-5D 3L, and loss of vertebral height; *p* < 0.025 for comparison of physical performance).

## Data Availability

The data associated with the paper are not publicly available but are available from the corresponding author upon reasonable request.
